# Monitoring processed, mature Human Immunodeficiency Virus type 1 particles immediately following treatment with a protease inhibitor-containing treatment regimen

**DOI:** 10.1186/1742-6405-2-2

**Published:** 2005-04-12

**Authors:** Heather A Baird, Andre J Marozsan, Michael M Lederman, Alan Landay, Donna Mildvan, Daniel R Kuritzkes, Harold A Kessler, Eric J Arts

**Affiliations:** 1 Division of Infectious Disease, Department of Medicine, Case School of Medicine and the Center for AIDS Research, Case Western Reserve University, University Hospitals of Cleveland, Cleveland, OH, USA; 2 Department of Pharmacology, Case School of Medicine, Case Western Reserve University, Cleveland, OH, USA; 3 Department of Immunology and Microbiology, Rush-Presbyterian-St. Luke's Medical Center, Chicago, IL, USA; 4 Division of Infectious Diseases, Beth Israel Medical Center, New York, NY, USA; 5 Harvard Medical School, Brigham and Women's Hospital, Boston, USA; 6 Section of Infectious Diseases, Department of Medicine, Rush-Presbyterian-St. Luke's Medical Center, Chicago, IL, USA

**Keywords:** Protease inhibitors, HIV-1, p24 antigen capture

## Abstract

Protease inhibitors (PIs) block HIV-1 maturation into an infectious virus particle by inhibiting the protease processing of gag and gag-pol precursor proteins. We have used a simple anti-HIV-1 p24 Western blot to monitor the processing of p55^gag ^precursor into the mature p24 capsid immediately following the first dosage of a PI-containing treatment regimen. Evidence of PI activity was observed in plasma virus as early as 72 hours post treatment-initiation and was predictive of plasma viral RNA decrease at 4 weeks.

## Background

Assembly and transport of the 55 kDa gag (p55^gag^) and 160 gag-pol (p160^gag-pol^) proteins to the inner plasma membrane is essential for the packaging of the viral genomic RNA, host tRNA^Lys,3 ^primer, as well as for interactions with HIV-1 envelope glycoproteins [5]. Budding and virus release initiates the processing of the *gag *and *gag-pol *precursor proteins. This processing step likely requires the dimerization of two *gag-pol *precursors (at least in the region of protease) that permits a low-efficiency cleavage of the precursor proteins and release of fully active protease (PR) homodimers [16]. These enzymes then complete protein maturation to produce an infectious virus particle. Thus, protease inhibitors (PI) appear to be most active at blocking HIV-1 replication following budding of the immature virus particle [4,6]. In contrast other antiretroviral drugs (ARV) such as nucleoside reverse transcriptase inhibitors (NRTI) and non-nucleoside RT inhibitors (NNRTI), block reverse transcription during intracellular HIV-1 replication [3].

To date, the best method to monitor inhibition of HIV-1 replication is to evaluate virus concentrations in the plasma [10]. Several commercial, FDA-approved assay kits (HIV-1 Quantiplex (bDNA) assay, AMPLICOR HIV-1 MONITOR assay, NucliSens HIV-1 assay) involve measuring virus levels via reverse transcription-PCR amplification of genomic HIV-1 RNA [8]. It is important to recognize however, that these assays cannot monitor the pharmacodynamic properties of many antiretroviral agents immediately following treatment initiation. Protease inhibitors block HIV-1 protease processing following virus release from cells in contrast to NNRTIs or NRTIs that inhibit during an intracellular replication step, i.e. reverse transcription. The half life of plasma virus is estimated to be approximately 6 hrs [13,18]. but the half life of activated CD4+ cells infected with and producing HIV even in the presence of PIs is approximately 1.2 days [13,18,19] during phase I decay An assay measuring levels of viral RNA does not distinguish between the immature virus (processing blocked by PIs) and infectious virus, both of which encapsidate HIV-1 genomic RNA. The estimated time required for protease inhibitors to clear the majority of free virus particles from the circulation and activated cells (not latently infected or quiescent cells) is approximately 4 weeks. Thus, a viral RNA assay performed on plasma does not provide a complete assessment of PI activity for at least 1–4 weeks.

## Methods

In this study, we tested the ability of three different assays to measure the quantity of both infectious virions and defective/immature virus particles in the plasma of HIV-infected patients who started treatment with a PI-containing regimen. The performance of three assays was validated in vitro utilizing HIV-1 infected cell lines in the presence or absence of PIs and other ARVs. These tests were followed by in vivo analyses using plasma samples from patients receiving a PI-based treatment regimen. The following provides a brief summary of the first two assays that could detect the effects of PI activity in vitro but failed in vivo.

The first assay involved measurement of infectious virus potential. We serially diluted cell-free culture supernatants from chronically HIV-infected U87.CD4.CCR5 cells treated with PIs. This diluted and undiluted plasma was then added to uninfected peripheral blood mononuclear cells (PBMC). Although this assay could be used to measure infectious potential of high titer viruses in tissue culture, only plasma containing extremely high viral loads (> 10^4 ^viral RNA copies/ml) could support any HIV-1 infection of PHA/IL-2 treated PBMC regardless of the patients treatment status (data not shown). Concentrating the virus by ultracentrifugation did little to increase infectious titer of virus from plasma.

The second assay involved PCR amplification of strong-stop viral DNA found in cell-free virus. Previous reports have shown that viral DNA is found HIV-1 particles [9,17] but that steric hindrance or the lack of dNTP substrates limit reverse transcription and presence of viral DNA to 1:1000 to 1:10,000 virions [1,2]. We have shown that a defective protease abolishes the synthesis of any HIV-1 DNA in virus particles [1,2]. HIV-1 strong-stop DNA was not detected by PCR amplification in virus produced from the chronically infected cells in the presence of PIs (data not shown). However, viral loads of >10,000 RNA copies/ml were required in patients to even detect the presence of HIV-1 DNA in plasma, which is consistent with previous findings. Thus, this assay was not effective for those patients starting PI therapy with lower viral loads (<10^3–4 ^RNA copies/ml).

In contrast to the assays described above, an anti-p24 Western blot was successful in measuring both in vitro and in vivo PI effects and was the simplest in design and application. To initially test this assay we infected U87.CD4.CXCR4 cells with a wild type HXB2 virus or the protease inhibitor resistant virus, RF (containing PR mutations V82F and I84V) [12]. Following established infection and stable virus production over three days (as measured by RT activity in the culture supernatant), cultures were treated with 0.2 and 20 nM lopinavir (LPV) or 2 and 200 nM nevirapine (NVP). The higher concentration of each drug was approximately 100-fold greater than the reported IC_50 _values (i.e. lower concentrations of each drug) [11,14,15]. Cell free culture supernatant (1 ml) was then harvested at 0, 4, 8, 24, and 72 h post drug addition. Virus was pelleted from the supernatant by ultracentrifugation (35,000 g for 1 h) and then resuspended in 50 μl of sodium-dodecyl sulfate (SDS) lysis buffer (1% SDS, 10% glycerol, 10% β-mercaptoethanol, 0.04 M Tris pH 6.8); of which 10 μl were heated to 95°C, separated on Tris-HCl-12.5% polyacrylamide precast gels (Bio-Rad), and transferred onto polyvinylidene difluoride membranes (Immobilon P; Millipore)by electroblotting (BioRad). Membranes were incubated with blocking reagent (5% milk-0.05% Tween in phosphate-bufferedsaline) for 1 h at room temperature then hybridized with a mouse anti-p24 monoclonal antibody (diluted 1:1,000; Fitzgerald Industries International, Inc.) overnight at 4°C. After washing, membranes were incubated with horseradish peroxidase-conjugated goat anti-mouse IgG1 antiserum (diluted 1:40,000; Pierce) for 3 hours. Immune complexes were visualized with the ECL system (Amersham) according to the manufacturer's instructions and films were analyzed using BioRad Quantity One software.

## Results and discussion

**Figure 1:**
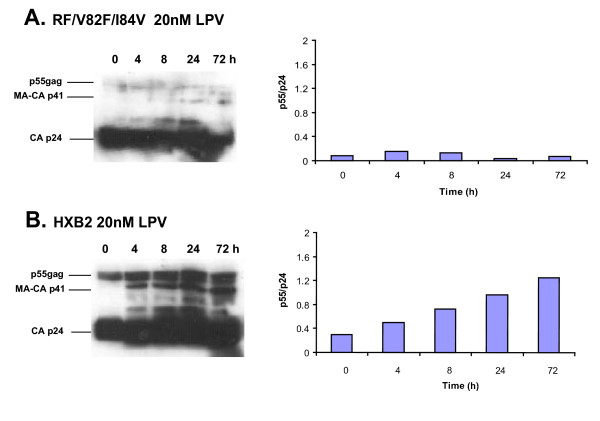
**Western blots for the HIV-1 *gag *proteins in HIV-1 produced in tissue culture following treatment with protease inhibitors. **U87/CD4/CXCR4 cells were plated in 6 well plates at 80,000 cells/well and allowed to grow to confluence. The cells were infected with either HXB2 or RF/V82F/I84V (protease inhibitor resistant virus) and RT activity was monitored. On day 3 of culture, infected cells were treated with 20 nM LPV, and 1 ml of media was removed at 0, 4, 8, 24, and 72 hours post-drug treatment. The virus was pelleted, and the pellet was then lysed using sodium-dodecyl sulfate (SDS) lysis buffer and then run on a 10% SDS polyacrylamide gel. Following transfer to nylon membranes, blots were probed with the primary mouse anti-p24 antibody and the horseradish peroxidase-conjugated goat anti-mouseantiserum. Films were exposed following treatment with the ECL kit (panel **A**). Ratio of unprocessed p55^gag ^to processed CA p24 over a 72 hour time course was determined by scanning the blots and quantifying the bands (panel **B**).

Fig. 1, panels A and B show Western blot analyses and p55:p24 ratios of two viruses grown in tissue culture, RF (protease inhibitor resistant) and HXB2 (protease inhibitor sensitive) and treated with 20 nM LPV. Two major bands appeared on the film of the ECL blot. The faster migrating product was the processed capsid (CA) p24 and the slower migrating band was unprocessed p55^gag^. We observed minute amounts of partly cleaved *gag *product containing the matrix (MA) p17 and CA p24, i.e. 41 kDa. In these experiments, the HXB2 p55:p24 is increasing over the first 72 hours of 20 nM LPV treatment indicating an inhibition of gag processing. In contrast, there was no evidence of decreased gag precursor processing with the RF virus treated with 20 nM LPV as indicated by a constant p55:p24 ratio of 0.1 during the 72 h of treatment. Treatment with a lower concentration of LPV (0.2 nM) or with NVP (2 or 200 nM) did not result in a significant difference in the HXB2 or RF p55:p24 ratios over time (data not shown). In tissue culture, longer incubation times with LPV (20 nM) reduced virus production to levels at which the quantities of p55 and p24 products were difficult to detect by Western blot analyses.

To test the utility of this simple Western blot assay to monitor initial PI treatment, nine patients were enrolled into the A5036s Substudy of the ACTG Clinical Trial Substudy A5014 [7]. All of the patients in A5014 were ARV treatment naïve and were randomized to receive either LPV+ the non-nucleoside RT inhibitor NVP or NVP + three nucleoside RT inhibitors: lamivudine (3TC) + stavudine (D4T) + abacavir (ABV). All participants and investigators in this study were blinded to the treatment arms. Ten ml of blood was drawn into Acid Citrate Dextrose (ACD) tubes prior to the first drug administration, then 4, 8, 12, 24, 72 hours, three days and four weeks post drug administration. Two aliquots of 3.5 ml of plasma were shipped on dry ice to CWRU and then stored at -70°C prior to analyses.

In addition to the Western blot analyses, the sub-study also called for a measure of infectious potential by HIV-1 found in plasma. For these tests we exposed HIV-negative peripheral blood mononuclear cells to plasma samples obtained prior to and immediately following treatment with the PI- or non PI-containing HAART regimen. Only plasma samples of one patient (of 9) resulted in productive infection of PHA/IL-2 treated PBMC cultures suggesting that this not a sensitive assay. Unfortunately, no assessment of PI activity could be evaluated using this infectious assay since this patient was treated with NVP and the three NRTIs. It is unlikely that viral levels in plasma is the sole factor contributing to the ability of plasma virus to infect PBMC cultures since all patients in this substudy had viral RNA loads approximately 10^4 ^copies/ml at initiation of treatment. The level of virus production or success of PBMC infections did not increase if the virus was concentrated from plasma by ultracentrifugation. This concentration step would also remove any residual drug in plasma that might affect infectivity of virus in plasma after the initial treatment.

**Figure 2:**
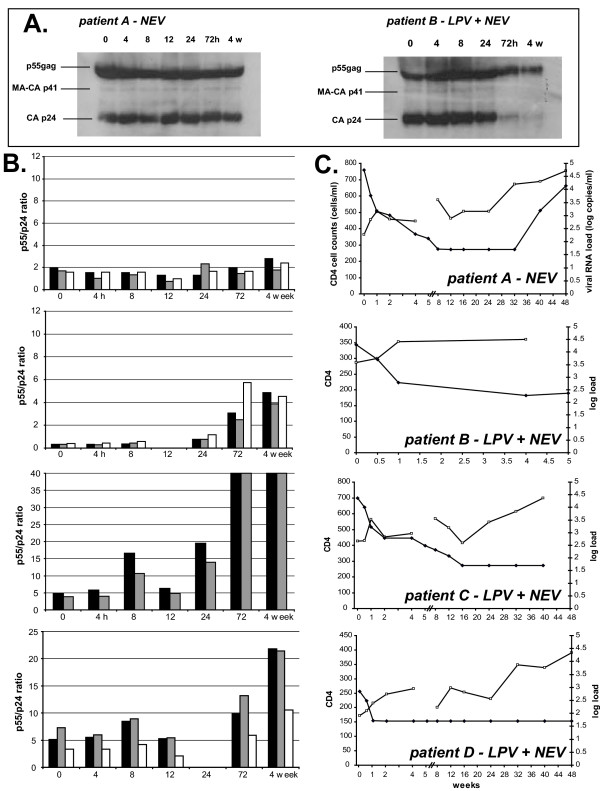
**Western blot analyses for the HIV-1 *gag *proteins in patient plasma prior to and following ARV treatment. **Patient plasma was obtained at 0, 4, 8, 12, 24, 72 hours and 4 weeks following ARV treatment. Plasma was diluted with serum-free media and then centrifuged to pellet HIV-1 particles prior to the analyses (see Fig. 1). Panel **A **shows the Western blot results on plasma samples obtained from patient A who was treated with NVP+3TC+D4T+ABV and patient B who was treated with LPV+NVP. Panel **B **shows the ratios of unprocessed p55^gag ^to processed CA p24 in patients treated with NVP+3TC+D4T+ABV (patient A) or NVP+LPV (patient B, C, and D). Each bar at each time point represents analyses from a separate Western blot. Panel C is a plot showing the changes in CD4 cell count (cells/mm^3^; open squares) and viral RNA load in plasma (copies/ml, filled diamonds) following treated with either treatment regimen.

Preliminary data indicated that plasma protein concentrations were too high to efficiently concentrate virus and resulted in excessive background on the Western blot for HIV-1 gag proteins. Thus, 1.5 ml of each plasma sample was diluted with 3.5 ml of phosphate-buffered saline (PBS) prior to concentrating the virus by ultracentrifugation. The procedures for the Western blot analyses are described above. Fig. 2A shows the Western blot results employing samples from patients A and B from the ACTG5014 clinical trial. It is important to note that this was a double-blinded trial and that all samples from each patient were analyzed prior to knowledge of treatment regimens [7]. Interestingly, the ratio of p55:p24 was greater than 1 in 8 of 9 patient samples prior to ARV treatment. High p55:p24 ratios suggest an increased proportion of noninfectious virus particles to infectious virions in the plasma. The ratio of p55:p24 in HIV-1 propagated in tissue culture is typically much less than one, suggestive of higher proportions of infectious to non-infectious virus in plasma. Most plasma proteins or free viral proteins were separated from the virus via centrifugation and pelleting of the virus. A 95% reduction of Coomassie blue staining of all proteins on the SDS PAG following transfer suggested that both the p55 and p24 proteins were efficiently electrotransferred to the nylon membranes. Increased ratios of p55:p24 was not due to selective antibody binding to the p55^gag ^considering the anti-p24 antibody should bind at least as efficiently to CA p24 than to the unprocessed p55^gag^. It is also possible that background p55 is due to the rapid turnover, and therefore that nascent virions make up a large fraction of the total. These virions could be more infectious than the fully processed ones seen in cell culture, since they would be rapidly processed in the course of the assay.

We predicted that the p55:p24 ratio would increase during first three days of PI treatment with the possible dips in this ratio between PI dosages (every 12 h). Preliminary data with patients starting a PI-containing treatment regimen suggest a PI-mediated inhibition of p55 processing within 8–12 h of treatment (data not shown). However, these studies were performed with patients starting RIT+SAQ or IND-containing treatment regimens and not with patients treated with LPV. As indicated by the results of tissue culture infection experiments shown in Fig. 1, the p55:p24 ratio should remain stable in plasma samples obtained from patients receiving non-PI containing HAART regimens (i.e. NVP+3TC+D4T+ABV) since neither NRTI nor NNRTI inhibit processing of the *gag *or *gag-pol *precursors.

Although only one example of these analyses is shown (Fig. 2B, panel I and II), the Western blot results of plasmas from each of four patients treated with the NVP+3TC+D4T+ABV combination showed a constant ratio of p55:p24 following treatment. In patients randomized to receive the LPV+NVP regimen, the ratio of p55:p24 increased at 72 h following the initial dosing (Fig. 2B). This increase in the p55:p24 ratio was maintained after 4 weeks of PI treatment. Previous findings revealed that HAART resulted in a drop in RNA and plasma infectivity in one day [20], however, the efficacy of ARV treatment can be affected by factors such as drug concentrations, compliance, potency, and selection of ARV resistant quasispecies. Unfortunately, two of the patients randomized to receive the LPV+NVP combination dropped out of the 5036 sub-study prior to the 72 h sample collection, i.e. the time that is likely required to detect a LPV block on viral protein maturation. In one patient, the p24 band on the Western blot was below the limit of detection in all plasma samples. There was an apparent delay in LPV activity following treatment in vivo as compared to treatment in tissue culture (Figs. 1 and 2). A longer time was likely required to attain inhibitory concentrations in blood or other tissues whereas the effect of LPV on newly produced virus particles was immediate in tissue culture.

**Figure 3:**
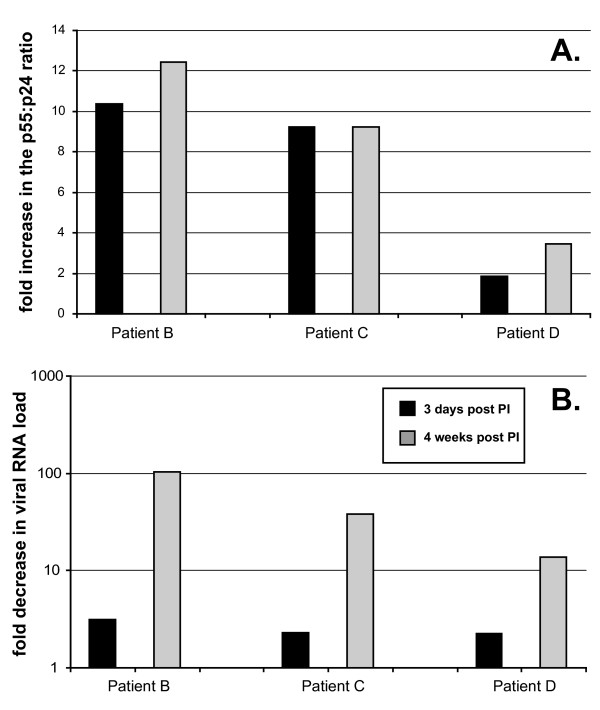
**Comparing the drop in viral RNA load to the increase in precursor gag protein following treatment with LPV and NVP. **The fold increase in the p55:p24 ratio was calculated by dividing these ratios at 3 days and 4 weeks by the observed ratio prior to treatment (time 0) (panel **A**). Panel **B **shows the fold decrease in viral RNA load at 3 days and 4 weeks following LPV+NVP treatment. This calculation involved dividing the viral load at day 3 and week 4 by that at time 0.

Nearly all antiretroviral drug-naïve patients recruited into AACTG 5014 demonstrate a drop in viral RNA load to undetectable levels after 8 weeks of treatment with either regimen. This viral load decrease was associated with an increase in CD4 cell counts. In patients B, C, and D, the drop in viral load was likely mediated by both NVP and LPV but the initial viral RNA load decrease (within one week) could be more of a measure of NVP than LPV inhibitory activity (Fig. 2C). Within three days, PI appeared to be blocking protease cleavage of precursor gag proteins in the virus particles found in plasma (Fig. 2B). This ratio increased only slightly during the next four weeks. Because this was a pilot study on a limited number of patients, it is difficult to ascertain what constitutes a significant change in p55:p24. However, it appears that there is a significant difference observed with the PI-containing regimen at 72 hours and 4 weeks. The minimal drop in viral load observed after three days post NVP+LPV treatment (<1 log) increased from a 13- to over a 100-fold decrease after four weeks of treatment (1.5 to 3 log decrease; panels II, III, IV in Fig. 2C and 3A). Interestingly, the relative decrease in viral load among these three LPV+NVP treated patients at four weeks also appeared to correspond to relative inhibition of protease cleavage at only three days post treatment (Fig. 3). Patient B showed a delayed and slower drop in viral load (at four weeks) and a higher increase in the p55:p24 ratio (at three days) than that observed in patient C and D (Fig. 3). A greater increase in p55:p24 ratio in patient B reflected the very low level of p55 detected. There was significant variation in the p55:p24 ratio (detected by Western blot) amongst all of the PI-naïve patients. Although difficult to test, this variation may be related to varying ratios of infectious virion:non-infectious virus particle production in HIV-infected individuals. These data suggest that the ratio of p55:p24 at three days following initiation of PI treatment may be predictive of the immediate HIV-1 inhibition by PIs in a patient. It should be noted that without the enrollment of more patients starting PI-based therapy, it is difficult to understand the relationship between the relative increase in the p55:p24 ratios and response to therapy except that the rapid increase in the ratio is strong indicator of anti-HIV PI activity in the patient.

## Conclusion

In summary HIV protease inhibitors block the processing of p55^gag ^and p160^gag-pol ^precursor proteins during virus budding or following virus release. However, the protease inhibitor does not impede incorporation of genomic HIV-1 RNA into the virus particle. Thus, following PI treatment, viral load assays based on detection of viral RNA measure both noninfectious, immature virus particles and virions found in plasma. We have developed a method to measure the anti-HIV activity of a protease inhibitor using a simple approach. In three patients treated with LPV+NVP, the ratio of unprocessed p55:processed p24 increased at 72 hours and over the next four weeks of treatment. In contrast, the ratio of HIV-1 p55:p24 did not change over the four weeks of study in patients treated with an NNRTI-containing regimen (NVP+3TC+D4T+ABV). This pilot study, though limited in patient number, has provided evidence that an HIV-1 p24 Western blot can be used to immediately measure the antiviral activity of protease inhibitors. Preliminary in vitro data also suggests that inability of PIs to block PI-resistant HIV-1 in patients could be assessed within 3 days of treatment. Based on these findings we are now testing the utility of this assay in highly PI experienced patients to predict the success of a new PI-containing treatment regimen within 3 days of starting this therapy. In addition, this study indicates that western blot is an excellent tool for the evaluation of the activity of protease inhibitors in vitro, and may be useful in evaluating new drugs putatively active against isolates resistant to current agents, or to evaluate the activity of different combinations of protease inhibitors using a more insightful measure than viral infectivity.

## Competing interests

The author(s) declare that they have no competing interests.

## Authors' contributions

H.B. and A.J.M. performed the laboratory work presented in this paper. E.J.A. supervised the laboratory work. M.M.L. and A.L. were the PI's of the parent study A5014. D.M. was the statistician for A5014 and 5036. D.R.K. was the protocol virologist for A5014.
